# Income-Based Disparities in Perceived Benefits and Challenges of Virtual Global Health Activities During the COVID-19 Pandemic: Mixed Methods Analysis

**DOI:** 10.2196/63066

**Published:** 2025-05-07

**Authors:** Shuo Zhou, Enid Kawala Kagoya, Alexandra Coria, Alyssa Beck, Jessica Evert, Marina Haque, Molly M Lamb, Amy RL Rule, Lisa Umphrey

**Affiliations:** 1 Department of Communication Studies School of Communication, AI & Media Psychology Lab, System Health Lab Hong Kong Baptist University Hong Kong China (Hong Kong); 2 Department of Community Health Institute of Public Health, Faculty of Health Sciences Busitema University Mbale Uganda; 3 Clinical Epidemiology Unit College of Health Sciences, School of Medicine Makerere University Kampala Uganda; 4 Department of Pediatrics Icahn School of Medicine at Mount Sinai New York, NY United States; 5 Department of Global Health and Health Systems Design Icahn School of Medicine at Mount Sinai New York, NY United States; 6 Mount Sinai Kravis Children's Hospital New York, NY United States; 7 Department of Epidemiology Colorado School of Public Health Aurora, CO United States; 8 Department of Family and Community Medicine University of California San Francisco, CA United States; 9 Child Family Health International El Cerrito, CA United States; 10 Department of Anesthesiology Wayne State University Detroit, MI United States; 11 Center for Global Health Colorado School of Public Health Aurora, CO United States; 12 Department of Pediatrics Emory School of Medicine and Children’s Healthcare of Atlanta Atlanta, GA United States; 13 Department of Pediatrics University of Colorado School of Medicine Aurora, CO United States

**Keywords:** global health, virtual, in-person, perceived benefits and challenges, COVID-19 pandemic, medical education

## Abstract

**Background:**

Global health activities (GHAs) can potentially reduce health disparities by facilitating resource sharing, promoting medical education and professional development worldwide, and enhancing collaboration among high-income countries (HICs) and low- and middle-income countries (LMICs). However, the COVID-19 pandemic disrupted in-person GHAs due to strict infection control and travel restrictions. To ensure the continuity of GHAs and further address health inequity, virtual GHAs (VGHAs) are gaining traction.

**Objective:**

Our research aimed to understand how people perceive the benefits and challenges of VGHAs, analyze and compare whether HIC and LMIC respondents have different perceptions of virtual and in-person GHAs, and summarize suggestions for improvement to inform the future development of VGHAs.

**Methods:**

We conducted a cross-sectional web-based survey during the COVID-19 pandemic in early 2022. Eligible participants were adult students, trainees, or professionals who participated in, created, taught, or facilitated GHAs. We thematically analyzed participants’ free-text responses regarding their perceptions of the benefits and challenges of virtual and in-person GHAs. The patterns differed depending on whether respondents were from HICs or LMICs; thus, we compared frequencies of each theme between the 2 groups.

**Results:**

A total of 154 respondents from 34 countries were included in the analysis. Key benefits of VGHAs were improved access to global health resources or content, reduced cost, easier scheduling and planning, expanded remote participation, and wider participation and reach. The themes that emerged as challenges of VGHAs included a lack of infrastructure to engage virtually, being less motivated and engaged, a lack of in-person and hands-on experience, and challenges with virtual communication and collaboration. LMIC respondents, compared to HIC counterparts, were more likely to identify reduced cost (26/67, 39% LMIC compared to 20/87, 23% HIC; *χ*^2^_1_=4.5; *P*=.03) and expanding knowledge, experience, or skills (15/67, 22% LMIC compared to 8/87, 9% HIC; *χ*^2^_1_=5.2; *P*=.02) as benefits of VGHAs, lack of infrastructure to engage virtually as a challenge of VGHAs (38/67, 57% LMIC compared to 31/87, 36% HIC; *χ*^2^_1_=6.8; *P*=.009), and to suggest improving the content to be more interesting and relevant (6/67, 9% LMIC compared to 1/87, 1% HIC; *χ*^2^_1_=5.3, *P*=.02). In contrast, HIC respondents were more likely to identify fostering continuity of relationship or activities (28/87, 32% HIC compared to 6/67, 9% LMIC; *χ*^2^_1_=11.9; *P*<.001) as a benefit of VGHAs and being less engaged and motivated to participate virtually (43/87, 49% HIC compared to 19/67, 28% LMIC; *χ*^2^_1_=7.0; *P*=.008) as a challenge of VGHAs.

**Conclusions:**

Our findings add to the existing literature by understanding how GHA participants from HICs and LMICs perceive the benefits and challenges of VGHAs differently. These data help elucidate what makes VGHAs acceptable to global health partners and suggest improvements to ensure partner needs are served equitably within the partnership.

## Introduction

### Background

Global health activities (GHAs) aim to advance international and interdisciplinary health care, research, education, public health, and policy, particularly focusing on promoting health equity worldwide [1-4]. These activities typically take place through institutional partnerships that can encompass a wide range of initiatives, including health care provision, collaborative research, health education, capacity building, access to technical expertise, resource sharing, and professional mentorship [5-8]. However, because true bidirectional exchange of people and resources is rarely achieved, and because of historical inequities in global health (GH) that disproportionately affect those in resource-constrained settings [9-12], GHAs can be inequitable regarding experience and opportunity for all partners [1,5,13-18] and can paradoxically exacerbate power differentials [7,15,18-21].

The COVID-19 pandemic widened and exposed existing health disparities globally and challenged countries’ capacities to cope with health crises. At the same time, GH partnerships played a critical role in facilitating the rapid exchange of information worldwide [22]. In response to the COVID-19 pandemic–related limitations on travel and the continued urgent need for collaboration, many GH partnerships pivoted to virtual GHAs (VGHAs). The increased use of virtual spaces could potentially address concerns around equity in GHAs [23,24]. However, studies of VGHAs have demonstrated that geographical and resource-level disparities also impact the way members of GH partnerships use and access them [23,24].

To address these challenges and build more equitable GH engagement practices and partnerships [19,20,25,26], it is critical to understand whether VGHAs are acceptable to diverse members of GH partnerships, the barriers to implementing VGHAs in various resource contexts, and how VGHAs may strengthen or weaken GH collaborations. Gaining this understanding will enable GH partners to effectively address previous partnership challenges [16,18,21,24,27], better capitalize on virtual opportunities for collaboration, and promote equity within partnerships [28,29]. All these improvements collectively can serve to address the inequities often inherent in GH practices, which contribute to and perpetuate power imbalances, unidirectional priority setting that do not favor the partners in low- and middle-income countries (LMICs), decision-making without key stakeholders present for discussions, and unethical imbalances in GH collaborations [9-12].

### Objective

To fill this knowledge gap, we conducted a mixed methods, cross-sectional web-based survey to gain insights from members of GH partnerships about in-person and virtual GHAs. This study examined participants’ perceived benefits and challenges of GHAs through a qualitative thematic analysis. We hypothesized that while LMIC and high-income country (HIC) respondents may have addressed similar themes, their emphasis on these themes may differ; therefore, we also present the results of a quantitative analysis comparing the theme frequency between these 2 groups.

## Methods

### Study Overview

We conducted a cross-sectional web-based survey to understand the perceived benefits and challenges of GHAs, identified differences in perceptions of GHAs between HIC and LMIC respondents, and derived suggestions for improving the quality of VGHAs. To compare people’s perceptions of virtual versus in-person GHAs, we asked the same open-ended questions for both types of activities. In this way, we could also include perspectives from participants who may not have personally had access to VGHAs, which nonetheless could inform the design of future VGHAs by considering the advantages and limitations of in-person GHAs. The qualitative analysis then gave rise to a secondary analysis to explore whether and how frequencies of various themes differed between respondents from HICs and LMICs.

### Participant Recruitment

The web-based questionnaire was open from January 18, 2022, to February 14, 2022. Eligible participants were professionals, students, or trainees of any health care–related discipline aged ≥18 years who facilitated, taught, created, or participated in in-person or virtual GHAs (including local or international clinical, public health, research, community, policy, and educational or development activities) either through an institution or as an individual. Multimedia Appendix 1 provides more detailed definitions of GHAs and related terminology. Participants were recruited using direct emails and listserves through purposive and snowball sampling strategies. We distributed the study information through authors’ professional networks, sending email invitations to 798 target participants who previously engaged with in-person GHAs and may have had some experience in VGHAs. We sent reminder emails twice during the data collection period to encourage participation. In the email, we included a standard study information leaflet with a notice of informed consent, study contact details, and a survey link. Potential respondents answered a set of screening questions; if eligible, they proceeded to the survey. Invitees from our target group further disseminated the web-based survey through their own networks. The study information was also shared on 2 large GH listserves.

### Ethical Considerations

We obtained ethics approval from the Colorado Multiple Institutional Review Board (University of Colorado, Aurora, CO, United States; 21-3020). We collected and stored data via the encrypted web-based survey platform REDCap (Research Electronic Data Capture; Vanderbilt University) [30]. Every participant consented to participate in this study. We deidentified all survey responses. Participants who completed the survey were eligible to enter a raffle to win a US $20 Amazon gift card.

### Survey Development and Measures

This study is part of a larger research project aimed at understanding participants’ access, interest, and equity considerations for VGHAs during the COVID-19 pandemic [31]. The survey contained both close-ended and open-ended questions based on a systematic literature review [23] and informed by findings from an exploratory project [24] on VGHAs. The full questionnaire is available in Multimedia Appendix 2. This study focused on participants’ responses to open-ended questions.

In free-text fields, we asked respondents to identify the main benefits and challenges of the virtual or in-person GHAs in which they had previously participated. We also asked their opinions on ways to improve the quality of VGHAs. All questions and answers were available in the English language.

### Analytical Approach

Using an inductive approach, we analyzed free-text responses to open-ended questions about the perceived benefits and challenges of virtual and in-person GHAs and how to improve VGHAs. We coded responses using Dedoose (version 9.0.46). To generate initial codes, 3 authors first familiarized themselves with the entire dataset and read the same responses from 15 randomly selected participants among the 154 participants (9.7% of the total sample). During this open-coding process, each coder highlighted relevant text and independently generated initial codes by identifying, categorizing, and labeling concepts related to the benefits and challenges of in-person and virtual GHAs and suggestions for VGHAs. The 3 coders compared their initial codes, discussed and resolved any discrepancies, and reached a consensus on an initial code list. We created notes to explain the meaning and properties of each code. The coders then analyzed the remaining responses using the code list, created new codes when data did not fit existing codes, and continued to refine the code list. After all open-ended responses were coded, the coders examined all data within each code to validate the codes and combine or split them into subcategories if appropriate. The 3 coders then categorized codes into broader themes.

To further compare perceptions of GHAs among different GH partners, we conducted a quantitative analysis of theme frequency. We counted the number and percentage of participants who generated responses related to each theme, stratified by country of residence. Respondents’ country of residence was categorized as either an HIC or LMIC based on the World Bank 2022 fiscal-year classifications [32]. Pearson *χ*^2^ tests were performed using SPSS (IBM Corp) to examine whether frequency of identified themes varied by participants living in LMICs compared to HICs.

## Results

### Overview

**Figure 1:**
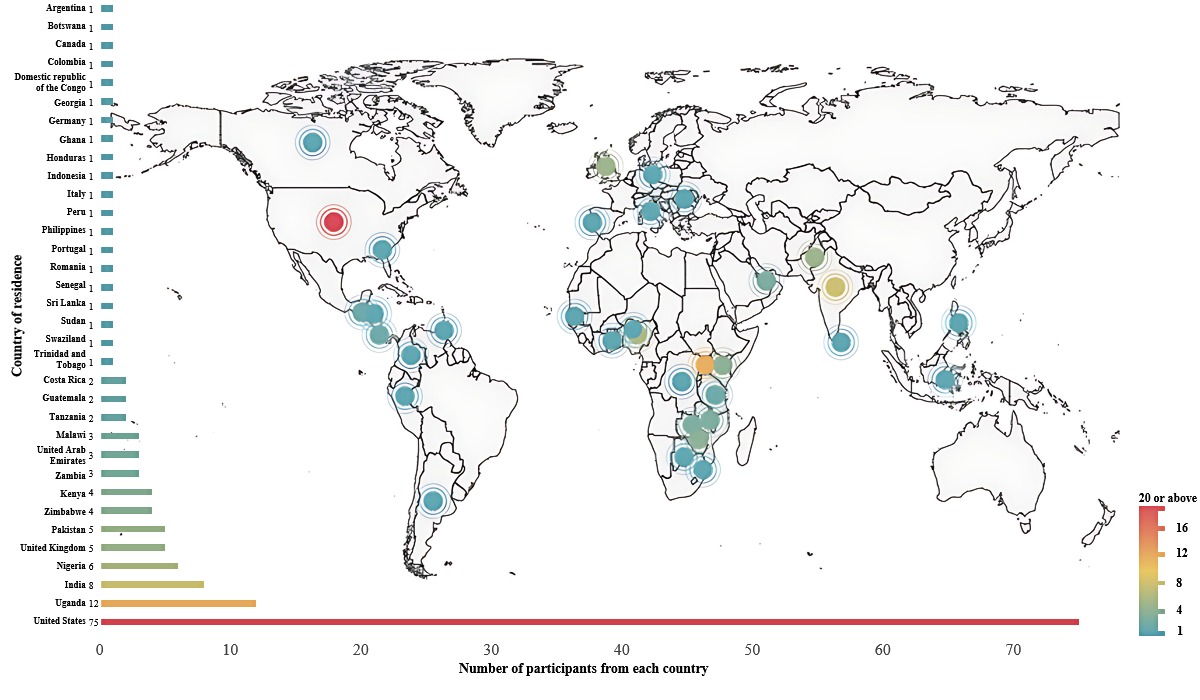
Distribution of respondents’ country of residence and the number of respondents from each country. See Multimedia Appendix 3 for a larger image.

A total of 347 respondents who completed screening questions were eligible for the study (response rate 43.5%), of whom 154 (44.4%) answered at least 1 open-ended question relevant to our research questions and thus were included in the analysis. Among the 154 respondents, 67 (43.5%) were from LMICs and 87 (56.5%) were from HICs, representing 34 countries (Figure 1, Multimedia Appendix 3). Table 1 summarizes the demographic and GH engagement characteristics of included respondents.

**Table 1:** Demographics and global health engagement characteristics of participants who responded to open-ended questions in the 2022 virtual global health activities international survey^a^.

Demographics			LMIC^b^ respondents (n=67), n (%)		HIC^c^ respondents (n=87), n (%)		Total respondents (n=154), n (%)
**Age (y)**							
	18-24	14 (20.9)		4 (4.6)		18 (11.7)	
	25-34	19 (28.4)		27 (31)		46 (29.9)	
	35-44	18 (26.9)		17 (19.5)		35 (22.7)	
	45-54	8 (11.9)		16 (18.4)		24 (15.6)	
	55-64	5 (7.5)		10 (11.5)		15 (9.7)	
	65-74	3 (4.5)		9 (10.3)		12 (7.8)	
	≥75	0 (0)		3 (3.4)		3 (1.9)	
	Prefer not to answer	0 (0)		1 (1.1)		1 (0.6)	
**Sex**							
	Male	32 (47.8)		29 (33.3)		61 (39.6)	
	Female	35 (52.2)		56 (64.4)		91 (59.1)	
	Other categories	0 (0)		1 (1.1)		1 (0.6)	
	Prefer not to answer	0 (0)		1 (1.1)		1 (0.6)	
**Education**							
	Completed primary school	0 (0)		0 (0)		0 (0)	
	Completed secondary school	2 (3)		0 (0)		2 (1.3)	
	Some university	13 (19.4)		1 (1.1)		14 (9.1)	
	Completed university	13 (19.4)		5 (5.7)		18 (11.7)	
	Some postgraduate	6 (9)		13 (14.9)		19 (12.3)	
	Completed postgraduate	33 (49.3)		68 (78.2)		101 (65.6)	
**Primary language**							
	Eastern Punjabi	1 (1.5)		0 (0)		1 (0.6)	
	English	50 (74.6)		82 (94.3)		132 (85.7)	
	French	2 (3)		1 (1.1)		3 (1.9)	
	Indonesian	1 (1.5)		0 (0)		1 (0.6)	
	Italian	0 (0)		1 (1.1)		1 (0.6)	
	Polish	1 (1.5)		0 (0)		1 (0.6)	
	Spanish	6 (9)		2 (2.3)		8 (5.2)	
	Swahili	1 (1.5)		0 (0)		1 (0.6)	
	Telugu	1 (1.5)		0 (0)		1 (0.6)	
	Urdu	2 (3)		0 (0)		2 (1.3)	
	Other	2 (3)		1 (1.1)		3 (1.9)	
**Primary position**							
	Academia or research	13 (19.4)		26 (29.9)		39 (25.3)	
	Administrative	2 (3)		9 (10.3)		11 (7.1)	
	Health professional	18 (26.9)		18 (20.7)		36 (23.4)	
	Organizational leadership	9 (13.4)		3 (3.4)		12 (7.8)	
	Public health or policy	7 (10.4)		3 (3.4)		10 (6.5)	
	Trainee	18 (26.9)		28 (32.2)		46 (29.9)	
**Clinical specialty**							
	Anesthesiology	0 (0)		1 (1.1)		1 (0.6)	
	Dentistry	1 (1.5)		1 (1.1)		2 (1.3)	
	Emergency medicine	4 (6)		8 (9.2)		12 (7.8)	
	Family medicine	7 (10.4)		4 (4.6)		11 (7.1)	
	Internal medicine	4 (6)		12 (13.8)		16 (10.4)	
	Mental health	1 (1.5)		1 (1.1)		2 (1.3)	
	Nursing	6 (9)		1 (1.1)		7 (4.5)	
	Pediatrics	5 (7.5)		29 (33.3)		34 (22.1)	
	Pharmacy	9 (13.4)		3 (3.4)		12 (7.8)	
	Surgery	3 (4.5)		2 (2.3)		5 (3.2)	
	Women’s health	1 (1.5)		1 (1.1)		2 (1.3)	
	In training	1 (1.5)		3 (3.4)		4 (2.6)	
	Other	5 (7.5)		4 (4.6)		9 (5.8)	
	Nonclinician	20 (29.9)		17 (19.5)		37 (24)	
**Engagement type**							
	Global health participant	25 (37.3)		23 (26.4)		48 (31.2)	
	Global health facilitator	14 (20.9)		34 (39.1)		48 (31.2)	
	Participant and facilitator	28 (41.8)		29 (33.3)		57 (37)	
	Do not know	0 (0)		1 (1.1)		1 (0.6)	

^a^Data are stratified by high-income and low- and middle-income classification as defined by country of residence.

^b^LMIC: low- and middle-income country.

^c^HIC: high-income country.

### Perceived Benefits and Challenges of In-Person GHAs

In total, 88.3% (136/154) and 82.5% (127/154) of participants described their perceived benefits and challenges of in-person GHAs, respectively. Multimedia Appendix 4 summarizes the main themes, representative quotes, and the number of HIC and LMIC respondents who expressed ideas under each theme.

The top benefit of in-person GHAs was *positive in-person experience* (90/154, 58.4%). Participants emphasized the importance of face-to-face interactions for enhancing peer-to-peer learning, understanding local contexts, gaining on-site experience, immersing in the culture, and engaging in hands-on practice. As one respondent noted:

There are so many things to learn from interacting with local providers in the countries that we visit, learning from their practice, creating that relationship, and working with their patient population. Although we are still able to cultivate relationships virtually, it is hard to deny the lessons learned by immersing oneself in a new culture and healthcare system in person.

Participant 132, HIC

Respondents also perceived *easier networking, new relationships, or activities* (65/154, 42.2%) as an advantage of in-person GHAs. Respondents explained the following:

Networking and working relationships are much improved with in-person experiences. So much more can be accomplished in Africa if it is in-person given the cultural differences.

Participant 138, LMIC

The top challenge of in-person GHAs was *high cost* (65/154, 42.2%), which included cost of travel, the need for accommodation, lack of or reduced GH funding due to the COVID-19 pandemic, and registration fees for activities. Another theme that emerged from participants’ responses was *COVID-19 pandemic-related concerns for engagement* (54/154, 35.1%), which was a unique challenge during the COVID-19 pandemic period given restrictions to in-person activities, strict travel and quarantine policies regulated by governments and health institutions worldwide, and people’s concerns about contracting SARS-CoV-2 while traveling. Respondents also perceived *required travel to site* (56/154, 36.4%) to be a huge commitment, involving travel-related time, effort, and financial and psychological burden.

### Perceived Benefits of VGHAs

In total, 97.4% (150/154) of respondents shared their thoughts regarding benefits of VGHAs. The primary perceived benefit of VGHAs was *improved access to GH resources and content* (101/154, 65.6%). Participants emphasized that it was easier to access resources at any time and from any place, particularly for people living in LMICs or remote areas. Virtual platforms increased the ability to share and disseminate GH resources, reuse or save GH content for future reference, and include a wider selection of materials. Some GHA facilitators also mentioned that the electronic form of GHAs improved their efficiency in developing GH resources as they could generate standardized training materials and make minor changes when necessary. Although accessing GH resources during the COVID-19 pandemic was challenging, virtual activities made it possible. As one participant stated, “[We are] unable to access [activities] in-person during the pandemic, so [virtual access has] been the only access at times” (Participant 19, HIC).

Another key benefit of VGHA was *reduced cost* (46/154, 29.9%). Respondents experienced reduced expenses mainly from 3 aspects: free or discounted fees to participate in VGHAs, free online GH resources and learning materials, and no travel or accommodation costs. As one participant said, “as based in Africa with financial problems, [accessing] free global health resources virtually was very beneficial for us” (Participant 97, LMIC).

Respondents also identified *enabling remote participation* in GHAs (43/154, 27.9%) as an advantage of VGHAs. Specifically, respondents perceived that attending GHAs virtually could avoid travel, which saved time, made the GH experience more efficient, was environmentally friendly, and improved safety during the COVID-19 pandemic period. Other themes related to the benefits of VGHAs are summarized in Multimedia Appendix 5.

### Perceived Challenges of VGHAs

In total, 93.5% (144/154) of respondents shared their perceived challenges of VGHAs. *Lack of infrastructure* (69/154, 44.8%) was the primary challenge of engaging with VGHAs. Participants mentioned poor or no internet connection, frequent power outages, lack of electronic devices, difficulties in charging their electronic devices, and lack of technical support to help them solve IT or connectivity-related issues. As one respondent described “bandwidth is very limited in rural settings which can limit meetings or even cancel or postpone them” (Participant 241, LMIC).

*Less engaged and motivated to participate virtually* (62/154, 40.3%) was another challenge of VGHAs. It specifically refers to the more granular challenges, such as Zoom burnout, web-based fatigue, and difficulty in concentrating during virtual activities. Respondents expressed that they were less motivated to participate in activities virtually and found virtual events less efficient than in-person interactions. One participant put it as follows:

There’s a lot of effort to make to remain focused as there’s always a distraction, especially when you have access at home when your kids are around. We also don’t feel motivated.

Participant 97, LMIC

The third key challenge was *lack of in-person and hands-on experience* (65/154, 42.2%). Participants mentioned that although virtual activities are helpful, the in-person connection was irreplaceable and that virtual activities did not provide the same benefits regarding hands-on experience, on-the-ground perspective, relationship building, and understanding of local needs. One participant pointed out the following:

For me, it lacks elements of the full experience. You cannot gauge the full picture from what is going on as you can when working side by side or being able to have social gatherings. Trying to have a detailed discussion about a topic online almost feels like an inquisition.

Participant 93, HIC

Another participant expressed the following:

The main challenge with virtual access is not being able to engage directly with colleagues. Virtual meetings are helpful, but cannot replicate the connections made with in person meetings.

Participant 58, LMIC

Other key themes emerged as the challenges of VGHAs included *difficult scheduling or planning* (45/154, 29.2%), which was mainly related to time zone differences and conflicting priorities, *difficult to network or build new relationships virtually* (43/154, 27.9%), and *challenges with virtual communication* (26/154, 16.9%), including greater misunderstanding and difficulty in interpreting accents and dialects without in-person explanations. Although not frequently mentioned, participants also pointed out *reduced knowledge or skills learned* (4/154, 2.6%) as the limited affordances of virtual platforms constrained learning for complicated procedures and simulations.

### Suggestions for Improving the Quality of VGHAs

Among all respondents, 36.4% (56/154) of participants provided 61 unique suggestions for improving the quality of VGHAs, which were categorized into 8 key themes (Multimedia Appendix 6). The most mentioned theme was to *make participation as easy and equitable as possible* (14/154, 9.1%). Participants suggested ensuring access to VGHAs across resource levels, improving the visibility of VGHAs by sharing the information via social media and various research networks, making the virtual platforms more user-friendly and less expensive, providing social activities and networking opportunities for people to make connections in a less formal and more meaningful way, and considering translation of materials into languages other than English for better accessibility of VGHAs.

Respondents also recommended that organizers of VGHAs *consider resource availability* (11/154, 7.1%), including addressing issues of internet connection, power shortage, lack of equipment, and building up basic infrastructure needed for virtual activities in LMIC settings.

The third most frequently mentioned suggestion was to *invest in training for VGHAs* (9/154, 5.8%). Such training should focus on educating both trainers and trainees on how to use technology. For example, it would be useful to provide workshops or training on how to use video conferencing tools, virtual platforms, mobile apps, and enhancing digital literacy.

Additional suggestions included *providing guidelines for VGHAs and better engagement* (8/154, 5.2%), such as standardizing GH curriculum and assessments of GH learning and sharing strategies to improve the engagement of GHAs; *making content as interesting and relevant as possible* (7/154, 4.5%), specifically providing more practical knowledge to LMIC participants and keeping GH materials up-to-date; *combining virtual with in-person activities* (4/154, 2.6%), such as providing more hybrid opportunities and linking virtual activities to future in-person activities; *improving institutional support and recognition of VGHAs* (4/154, 2.6%); and *pursuing collaborations for VGHAs* (3/154, 1.9%), especially creating and expanding collaboration opportunities with low-income countries.

### Comparing Perceptions Between LMIC and HIC Respondents

A quantitative analysis compared whether LMIC and HIC respondents differed in their perceptions of in-person and virtual GHAs. Multimedia Appendices 4-6 demonstrate percentages of participants from the 2 groups mentioning each theme. We found that a greater proportion of LMIC respondents compared to HIC respondents identified *reduced cost* (26/67, 39% LMIC compared to 20/87, 23% HIC; *χ*^2^_1_=4.5; *P*=.03) and *expanding knowledge, experience, or skills* (15/67, 22% LMIC compared to 8/87, 9% HIC; *χ*^2^_1_=5.2; *P*=.02) as the benefits of VGHAs. Regarding perceived challenges of VGHAs, LMIC respondents identified *lack of infrastructure to engage virtually* (38/67, 57%; *χ*^2^_1_=6.8; *P*=.009) and *high cost to participate and lack of funding* (13/67, 19%; *χ*^2^_1_=5.5; *P*=.02) more frequently than HIC respondents (31/87, 36% and 6/87, 7%, respectively). Regarding recommendations for improving VGHAs, LMIC respondents (6/67, 9%) were more likely than HIC respondents (1/87, 1%) to emphasize *making content as interesting and relevant as possible* (*χ*^2^_1_=5.3, *P*=.02).

In contrast, greater percentages of HIC respondents than LMIC respondents identified *fostering continuity of relationship or activities* (28/87, 32% HIC compared to 6/67, 9% LMIC; *χ*^2^_1_=11.9; *P*<.001) as the benefit of VGHAs; *less engaged and motivated to participate virtually* (43/87, 49% HIC compared to 19/67, 28% LMIC; *χ*^2^_1_=7.0; *P*=.008) as the challenge of VGHAs; *positive in-person experiences* (60/87, 69% HIC compared to 30/67, 45% LMIC; *χ*^2^_1_=9.1; *P*=.003), *easier networking, new relationships, or activities*, (45/87, 52% HIC compared to 20/67, 30% LMIC; *χ*^2^_1_=7.4; *P*=.007) and *improved interest or engagement in GH* (14/87, 16% HIC compared to 3/67, 4% LMIC; *χ*^2^_1_=5.2; *P*=.02) as the benefits of in-person GHAs; and *difficulty in scheduling or planning* (26/87, 30% HIC compared to 11/67, 16% LMIC; *χ*^2^_1_=3.8; *P*=.05) as the challenge of in-person GHAs.

## Discussion

### Principal Findings

Our qualitative data describe what participants in GHAs find beneficial and challenging about both virtual and in-person GHAs, which is useful in optimizing the experience of VGHAs. However, participants from HICs and LMICs may view the importance of these challenges and benefits differently. This observation is critically important in the journey to mitigate power imbalances and historical inequities in GH partnerships [9-12]. Where partners’ challenges and benefits differ, the perspective, needs, and priorities of an LMIC partner are often overlooked in this partnership, for reasons related to funding and historically unethical structural inequities, unless the partners explicitly undertake work to ensure that challenges experienced by the LMIC partner are addressed and their needs are prioritized [9,23,33].

### Differences Between Perceptions From LMIC and HIC Partners

In our dataset, themes suggest mismatches in priorities, agenda setting, and equitable collaboration between LMIC and HIC partners. LMIC respondents identified a lower cost of VGHAs (*P*=.03) and the ability to expand knowledge, experience, and skills (*P*=.02) as benefits of virtual engagement significantly more often than their HIC colleagues. To explore how to prioritize these benefits, and in doing so center the needs of the LMIC partners, future research should focus on comparing costs and impact between virtual and in-person GHAs.

Conversely, HIC respondents identified the benefit of continuity of relationships significantly more often than LMIC counterparts (*P*<.001). This finding suggests that HIC participants are more likely to recognize the importance of maintaining GH relationships and benefit more from continued collaboration through virtual engagements. At the same time, LMIC participants may highly value activities that could help address immediate challenges rather than investment in long-term engagements. To foster equitable GH relationships, future VGHAs should consider the different priorities of LMIC and HIC participants and balance addressing immediate health challenges crucial for LMICs with recognizing the value of sustained collaborations appreciated by HICs.

Regarding the challenges of VGHAs, responses from LMIC participants more frequently reflected themes around the lack of technical infrastructure (*P*=.009) and high costs to participate or lack of funding (*P*=.02). The repeated mention of high costs and lack of technical support from LMIC respondents highlights a potentially surprising downside to virtual engagement meriting further evaluation within partnerships. Additional costs of VGHAs identified by our respondents included subscription fees for web-based content, registration fees for web-based conferences, and costs associated with technological and infrastructural upgrades to access reliable Wi-Fi and electricity. Furthermore, LMIC respondents mentioned the need for support or training on digital literacy to successfully participate on virtual platforms. These data add to previous studies describing the financial and technological barriers hiding beneath the surface-level ease of virtual engagement, challenges that may not be apparent to HIC partners requesting or offering virtual engagement to their LMIC colleagues [19,20].

HIC respondents reported being less engaged and motivated to participate VGHAs more frequently than LMIC respondents (*P*=.008). Further studies are encouraged to evaluate the “weight” of this challenge and its ultimate impact on the success of VGHAs. While the challenges described by HIC respondents are inconvenient at best and detrimental to partnerships at worst, the challenges described by LMIC respondents completely block participation in many LMIC settings without significant financial and infrastructural support and improvements. A frank cost-benefit and barrier analysis should become a routine part of every GH partnership when implementing new, or continuing with, virtual GH engagement.

### Suggestions for Improving the Quality of VGHAs

Respondents provided recommendations for improvements to VGHAs that focused on 8 key themes (Multimedia Appendix 6), which reveal important trends. LMIC respondents mentioned themes that reflected a lack of consideration, inclusion, and decision-making in their GH experiences. A greater percentage of LMIC respondents suggested that making GH content interesting and relevant to them would improve their experience (*P*=.02), which implies that current VGHA content may mainly be driven by HICs’ perspectives and benefit HIC respondents more. Other themes—need for better guidance and training for engagement (*P*=.07) and improving the ease and equity of participation (*P*=.10)—were also more common among LMIC respondents, although these differences did not reach statistical significance. Considering our other findings, these data continue to paint a picture of inherent inequity related to agenda and priority setting and decision-making.

Potential strategies to increase the equity of GHAs include (1) establishing standardized practices for GHAs and making them publicly available, to ensure that all partners understand the expectations; (2) providing regular orientations and training on technology literacy—such as how to use web-based platforms, video conferencing tools, and mobile apps—by partners more experienced in virtual education and technology; and (3) addressing financial barriers by rerouting funds previously earmarked for in-person GHAs.

Combining virtual with in-person GHAs moving forward is interesting as a potential best-practice recommendation for VGHAs. The incorporation of VGHAs to augment and prolong GH engagement is a promising strategy to improve equity in GH partnerships as it could lengthen opportunities for collaboration, open avenues of education, and bring to the table both sides of partnerships for participation in agenda setting and decision-making longitudinally.

Guided by the suggestions provided by respondents, in Textbox 1 we propose several recommendations for enhancing the quality of future VGHAs.

**Recommendations for virtual global health engagement.**

**To evaluate and address potential benefits**

- Before engaging in virtual global health activities (VGHAs), undertake a frank evaluation of both low- and middle-income country (LMIC) and high-income country (HIC) partners’ perceived benefits and priorities for virtual engagement.
- Identify the “weight” of each benefit for each partner.
- Undertake a comparison of expectations and benefits and ensure that they are equitable.
- When expectations and benefits are not equal between partners, prioritize the needs of LMIC partners.
- Conduct frequent evaluations of partnership activities to evaluate positive or negative experiences, address problems, and launch a new feedback cycle.
- Consider resource availability by addressing issues related to internet connection and building capacity.
- Consider investing in infrastructure and providing resources, such as internet access, laptops, and technical support for LMIC participants, ensuring that all partners have the resources necessary for meaningful engagement.

**To evaluate and remove potential barriers**

- Before engaging in VGHAs, undertake a frank evaluation of both LMIC and HIC partners’ perceived barriers to virtual engagement.
- Identify how each partner might be negatively impacted by engaging virtually.
- Identify the “weight” of each barrier for each partner.
- Undertake a comparison of barriers between partners.
- Evaluate all technological and infrastructural costs to engage in the VGHAs.
- Create a budget for each activity planned, with clear and transparent discussions about what is available to and covered by each partner.
- Identify action plans to address immediate challenges and more easily solved issues.
- Collaboratively create longer-term approaches for more systematic and resource-intensive solutions.
- Address knowledge gaps in virtual literacy through training and orientation sessions that are accessible and adaptable for all partners.
- Advocate heavily within HIC institutions for the reappropriation of global health (GH) funds toward rectifying barriers LMIC partners face for global health activity (GHA) engagement.

**To improve overall VGHA experiences**

- Advocate within HIC institutions for institutional support and recognition of GHA, both for their own and their partner’s contributions.
- Make GH content interesting and relevant to LMIC partners, providing hands-on practice whenever possible, codeveloping standardized curricula, and keeping materials up-to-date.
- Involve participants from both LMIC and HIC partnership sites in the planning process and seek their input to ensure that all partners have an equal voice and activities are convenient, feasible, and mutually beneficial for them.
- Improve guidance and training for engagement in virtual activities.
- Improve the ease of participation in VGHAs (such as by removing web-based barriers to content, considering time zone changes, and prioritizing the needs of LMIC partners in interactions).
- Consider resource availability for both LMIC and HIC partners when planning VGHAs.
- Improve collaborations within VGHAs to ensure both LMIC and HIC partners’ needs, priorities, and wishes are considered.

**To consider combining virtual with in-person GHAs**

- Consider hybrid models that offer both virtual and in-person options to increase flexibility and cater to the needs and preferences of diverse participants.
- When engaging in person, work ahead of time to jointly discuss priorities and agenda setting, ensuring an inclusive process that reflects all partners, especially LMIC partners’ perspectives.
- Conduct a needs assessment to identify the barriers partners must engage equitably.
- Seek to address short-term barriers with an actionable step-by-step plan, including deadlines.
- Consider the goal of the VGHA by matching it with the most suitable event format, maximizing the benefits of in-person or virtual activities.
- Collaboratively develop a plan for how to address more challenging barriers over the long term, always with priority settings made by the LMIC partner.

### Limitations

The results of this study should be interpreted with caution due to the following limitations. First, our analyses were based on participants who voluntarily responded to our survey and completed one or more open-ended questions. People who declined to express their perceptions about GHAs in free texts were not included, resulting in self-selection bias. Because of the nonrandom sampling strategy, these results may not be generalizable beyond the study population. Although we recruited clinical practitioners, medical students, and trainees focusing on various specialties, this survey may not reach enough frontline health professionals who worked closely with patients with COVID-19 or organizational leaders. This may result in our results not covering a full range of GH initiatives, particularly collaborative activities related to disease control and prevention and health care system strengthening. In addition, participants may suffer from recall biases and only mention the most salient GHAs, leading to a relatively narrow scope of GHAs discussed in this study. Future studies may recruit a more representative sample of GHA participants and facilitators to encompass a broader range of GH initiatives. Second, this cross-sectional survey was administered toward the end of the COVID-19 outbreak, we could not determine whether respondents’ perceptions were based on their experiences with virtual and in-person GHAs before or during the COVID-19 pandemic. Without specifying the time of their experience, we should not make inferences about specific perceptions of GHA changes during the COVID-19 pandemic. It is worth tracking and comparing people’s perceptions of GHAs and the income-based disparities before, during, and after the COVID-19 pandemic in future research. Third, while English is the primary language for most participants, respondents from LMICs and HICs varied in language skills. In general, participants from HICs wrote longer responses to the open-ended questions compared to their LMIC counterparts. Therefore, the themes, especially related to the benefits and challenges of in-person GHAs, were more frequently mentioned by HIC as compared to LMIC respondents. Future research projects may allow respondents to write in other languages. Fourth, we recognize that using LMIC or HIC classifications may not be nuanced enough to directly reflect differences in health resources and access [34,35]. Our conclusions may have been different had we used alternative classifications. In addition, income-based disparities may be analyzed based on different indicators. This study measured disparity in terms of the access and resources that people had based on their geographical site of work in an LMIC or HIC. Future studies are encouraged to measure individual participants’ income, social class, educational, and geographical background to have a more comprehensive understanding of these disparities.

### Conclusions

The findings of this mixed methods study enhance our understanding of perceptions of VGHAs and provide insights as to how to address current challenges and improve VGHAs. The significant differences and trends in our data suggest key mismatches between GH partners in LMIC versus HIC settings regarding benefits and challenges for VGHAs and suggestions for improvement. Despite some discrepancies in responses from LMICs and HICs, these findings add to the literature by highlighting the potential of VGHAs to significantly increase access to GH resources, expand reach to more participants, and provide opportunities for global collaboration despite physical distance and resource constraints. Given the potential financial and capacity-building benefits of VGHAs, they offer promise as tools to address the historical inequities in GH relationships.

## Appendix

Multimedia Appendix 1 Definitions of global health activities.

Multimedia Appendix 2 Questionnaire.

Multimedia Appendix 3 Distribution of respondents’ country of residence and the number of respondents from each country.

Multimedia Appendix 4 A summary of themes on perceived benefits and challenges of in-person global health activities stratified by low- and middle-income country and high-income country respondents.

Multimedia Appendix 5 A summary of themes on perceived benefits and challenges of virtual global health activities stratified by low- and middle-income country and high-income country respondents.

Multimedia Appendix 6 A summary of themes on suggestions for improving the quality of virtual global health activities stratified by low- and middle-income country and high-income country respondents.

## Abbreviations

GH: global health
GHA: global health activity
HIC: high-income country
LMIC: low- and middle-income country
REDCap: Research Electronic Data Capture
VGHA: virtual global health activity

## Data Availability

The data that support the findings of this study are available from the corresponding author upon reasonable request.
