# Supplementary material for: Income-Based Disparities in Perceived Benefits and Challenges of Virtual Global Health Activities During the COVID-19 Pandemic: Mixed Methods Analysis
Source: J Med Internet Res. 2025 May 7;27:e63066. doi: 10.2196/63066 (PMC12096022; doi:10.2196/63066)
Supplement: Multimedia Appendix 1 [file jmir_v27i1e63066_app1.pdf]

## Multimedia Appendix 1

### Definitions of Global Health Activities, Participants, and Facilitators

| | |
|---|---|
| Global Health Activity (GHA) | Any health activity focused on social accountability, equity, and cultural humility, which seeks to bridge geographical distance and/or resource levels. Activities are rooted in the collaborative, interdisciplinary practice of patient and population-centered healthcare and may focus on clinical, public health, research, community, policy, educational and/or development work. Further, activities may occur individually, between individuals, between organizations/institutions, or between individuals and organizations/institutions. |
| Global Health Participant | Any consumer of global health education materials or participant in global health activities, whether a student, post-graduate learner or adult learner pursuing continuing education |
| Global Health Facilitator | Any person who develops, facilitates, hosts, and/or provides global health education or activities to participants, either within one organization or within global health partnerships |
