# Supplementary material for: Income-Based Disparities in Perceived Benefits and Challenges of Virtual Global Health Activities During the COVID-19 Pandemic: Mixed Methods Analysis
Source: J Med Internet Res. 2025 May 7;27:e63066. doi: 10.2196/63066 (PMC12096022; doi:10.2196/63066)
Supplement: Multimedia Appendix 2 [file jmir_v27i1e63066_app2.pdf]

Multimedia Appendix 2

Virtual Global Health Activities (VGHA) International Survey Study

Specific Project Objectives:

1. To describe characteristics of respondents who participate in global health activities (GHAs)
2. To describe participants' global health activities: differences among respondent types, characteristics of their activities, in-person vs virtual nature of activities, and chronology of activities both before and during the COVID-19 pandemic
3. To determine perceived benefits and challenges of VGHAs
4. To determine participant interest in VGHAs, particularly those anticipated to endure after the pandemic
5. To identify potential geographic differences in preferred VGHAs

Acronyms

GH = global health
GHA = global health activity
VGHA = virtual global health activity

Definitions

Global health activities = any health activity focused on social accountability, equity, and cultural humility, which seeks to bridge geographical distance and/or resource levels. Activities are rooted in the collaborative, interdisciplinary practice of patient and population-centered healthcare and may focus on clinical, public health, research, community, policy, educational and/or development work. Further, activities may occur individually, between individuals, between organizations/institutions, or between individuals and organizations/institutions.

Global Health Partnership = any partnership between two or more individuals, institutions or organizations engaging in global health activities, as defined above

Collaborative global health activities = activities in which members of all sides of a global health partnership(s) are involved in the preparation or presentation of the activity, and ideally members from both/several sides of a partnership attend and actively participate in the activity in real time.

Global Health Education = any educational initiative focused on a global health activity, as defined above

Global health experiences or electives = engagement with global health electives, rotations, observer placements or other placements within or outside my organization's catchment area

Organization = where a participant works, undergoes training, or participates in global health activities

Global Health Participant = any consumer of global health education materials or participant in global health activities, whether a student, post-graduate learner or adult learner pursuing continuing education

Global Health Facilitator = any person who develops, facilitates, hosts, and/or provides global health education or activities to participants, either within one organization or within global health partnerships

Consent

You are being asked to be in this research study because you are a global health facilitator or participant involved with global health activities.

If you join the study, you will participate in a 10-15-minute survey.

This study is designed to learn more about how global health activities were affected by the COVID-19 pandemic, specifically on how activities have or may become virtual.

The researchers have not identified any possible discomforts or risks in taking this anonymous, online survey. There may be risks the researchers have not thought of.

Every effort will be made to protect your privacy and confidentiality by keeping your information confidential and locked in a password-protected database. The survey will not ask you to provide any personal, sensitive, or protected information.

You have a choice about being in this study. You do not have to be in this study if you do not want to be. If you start the survey, you may stop the study at any time.

If you have questions, you can email the study Principal Investigator.

You may have questions about your rights as someone in this study. If you have questions, you can call the COMIRB (the responsible Institutional Review Board) at +1 (303) 724-1055.

At the end of the survey, you will be given the option to participate in a raffle for one of five Amazon gift cards. We will not store or later access your personal information linked to this raffle.

Do you agree to participate in this survey?
Yes
No (**STOP survey if NO**) – *"Thank you for your time."*

Are you at least 18 years old?
Yes
No (**STOP survey if NO**) – *"Thank you for your time."*

Are you involved in global health activities as defined above *(including local or international clinical, public health, research, community, policy, educational and/or development activities)*?
Yes
No (**STOP survey if NO**) – *"Thank you for your time."*

Part A. Characteristics of respondents participating in global health activities (Addresses: OBJECTIVE 1 & 5)

1. Where do you currently live? (drop down menu - countries)

2. Where do you participate in most of your global health activities? You may pick up to 5 different countries. (*drop down menu – countries, choose one*) (allow 5 repeats to reflect up to 5 countries)

3. If you participate in most of your global health activities in a high-income country, is/are the activity/ies focused in a low-resource environment within that high-income country?
    a. Yes
    b. No
    c. Don't know

4. What is your age? (*drop down menu – age bracket by decade*)

5. With which gender do you identify? *(drop down menu – gender options)*

6. What is your primary language of work or education? *(drop down menu – language options)*

7. What is your highest degree completed? *(drop down menu – degree options)*

8. What is your current primary position? *(Choose one best option)*
    a. Administrator of hospital, health center or organization
    b. Administrator of academic training/teaching program (post-graduate, residency, fellowship, etc.)
    c. Clinical instructor
    d. Clinician, including advanced or midlevel practice provider
    e. Fellow
    f. Nurse
    g. Organization director / president / CEO / Executive Director
    h. Organization officer / manager
    i. Pharmacist
    j. Physical Therapist / Physiotherapist / Rehabilitation
    k. Policy maker or advisor
    l. Public health professional
    m. Professor / Lecturer
    n. Researcher
    o. Resident or chief resident
    p. Student, health professional (medical, nursing, pharmacy, etc.)
    q. Student, other
    r. Other (free response)

9. If you work or are training clinically, what is your specialty? *(Choose one best option)*
    a. N/A: I'm not a clinician or clinical learner
    b. Dentistry

c. Emergency medicine, general
d. Emergency medicine, pediatric
e. Family medicine or general practice
f. Internal medicine
g. Internal medicine subspecialist
h. Midwifery
i. Nursing
j. OB/GYN
k. Pediatrics
l. Pediatrics and Internal Medicine, combined
m. Pediatric subspecialist
n. Pharmacy
o. Surgery, general
p. Surgery, subspecialty
q. Other (free response)

Part B. Characteristics of respondents' global health activities
(Addresses: OBJECTIVE 1, 2 & 5)

*Remember that a **global health*** **participant** is any consumer of global health education materials or participant in global health activities, whether a student, post-graduate learner or adult learner pursuing continuing education

*Remember that a **global health facilitator*** is any person who develops, facilitates, hosts, and/or provides global health education or activities to participants, either within one organization or within global health partnerships

10. We would like to know the main way in which you engage in global health activities. Do you consider yourself more of a:
    a. Global health participant
    b. Global health facilitator
    c. Participant and facilitator
    d. Other *(Free text)*

11. Do you personally have access to an administrative support person who assists with your global health activities (i.e., for scheduling, logistics, editing, etc.)?
    a. Yes, full time support
    b. Yes, part time support
    c. No
    d. Don't know
    e. N/A

12. Do you personally have access to any of the following types of funding for your global health activities? *(check all that apply)*
    a. Philanthropic donations
    b. Grant funding
    c. My own organization's budget
    d. A partner organization's budget

e. Self-funded/personal funds
f. Medical/health professions tuition
g. I do not have access to global health funding
h. Don't know
i. Other (free text)

12.1 (Skip logic) If you have access to financial support, for what global health expenses are you permitted to use your funds? *(check all that apply)*

a. Education programs and/or activities at my organization
b. Global health travel
c. Conference expenses (hosting or attending)
d. Publication expenses
e. Salary support
f. Training supplies
g. Research support
h. CME/professional development
i. Don't know

12.2 (Skip logic) If you have access to financial support, how did the COVID-19 pandemic affect your access to funding?

a. I had better access to funding because of the pandemic
b. I had decreased access to funding because of the pandemic
c. I experienced no change in access to funding because of the pandemic
d. Don't know

12.3 (Skip logic) If you have access to financial support, is your global health funding in addition to your core role funding?

a. Yes
b. No
c. Don't know

Part C. Participant access to and interest in virtual global health activities
(Addresses: OBJECTIVE 2, 3 & 5)

Instructions:
Complete the following questions from your own perspective. If you are a global health facilitator and are not directly involved in the listed activities, respond in terms of participants you support. Please check an activity as 'during pandemic' if it started after March 2020, even if not ongoing.

13. Complete Part C-1

Part C-1: Access to global health activities and resources

I have/had access to (or participants at my organization have/had access to)… (check all that apply)

Activity	At my organization:				At another organization:				N/A (Check this box if you did not have access to the activity)	Don't know
	Pre-pandemic *(before March 2020)*		During pandemic *(after March 2020)*		Pre-pandemic *(before March 2020)*		During pandemic *(after March 2020)*			
	In person	virtual	In person	virtual	In person	virtual	In person	virtual		
professional resources (i.e., library services, access to journals/publications, online subscription services, etc.) that support global health activities										
global health educational materials (i.e., curricula, textbooks, modules, etc.)										
global health didactic sessions (i.e., grand rounds, case										

discussions or journal clubs, etc.)										
global health simulation sessions (i.e., clinical, procedural, or cultural simulations)										
I have participated in the **creation of global health materials**										

Note: The Following questions will be asked once per Part C subsection

We now will ask you about your general experience with **accessing global health resources.**

14. In your experience, what have been the main **benefits** of **accessing global health resources virtually?** *Please describe the benefits and explain why you think these are the main benefits for you. (free text or NA)*
15. In your experience, what have been the main **concerns and challenges** of **accessing global health resources virtually?** *? Please describe the concerns and challenges and explain why you think these are the main concerns and challenges for you. (free text or NA)*
16. In your experience, what have been the main **benefits** of **accessing global health resources in person?** *Please describe the benefits and explain why you think these are the main benefits for you. (free text or NA)*
17. In your experience, what have been the main **concerns and challenges** of **accessing global health resources in person?** *Please describe the concerns and challenges and explain why you think these are the main concerns and challenges for you. (free text or NA)*

18. Complete Part C-2

Part C-2: Global Health Experiences and Electives

Global health experiences or electives are defined as engagement with electives, rotations, or placements within or outside my organization's catchment area.

- Local experiences occur within the participant's home community
- Domestic/national experiences occur within the participant's home country
- International experiences occur outside the participant's home country

I have/had access to (or participants at my organization have/had access to)… (check all that apply)

Activity	**At my organization**				**At another organization**				**N/A** (Check this box if you did not have access to the activity)	**Don't know**
	**Pre-pandemic *(before March 2020)***		**During pandemic *(after March 2020)***		**Pre-pandemic *(before March 2020)***		**During pandemic *(after March 2020)***			
	**In person**	**virtual**	**In person**	**virtual**	**In person**	**virtual**	**In person**	**virtual**		
global health experience preparation sessions (i.e., pre-departure trainings, global health boot camps, cultural trainings, etc.)										
**local** global health experiences										
**Domestic/national** global health experiences										
**international** global health experiences										

Hosting of global health participants from outside my organization										

Note: The Following questions will be asked once per Part C subsection

We now will ask you about your general experience with **global health electives/experiences.**

19. In your experience, what have been the main **benefits** of **virtual global health electives/experiences?** *Please describe the benefits and explain why you think these are the main benefits for you (free text or NA)*
20. In your experience, what have been the main **concerns and challenges** of **virtual global health electives/experiences?** *Please describe the concerns and challenges and explain why you think these are the main concerns and challenges for you.*
21. *(free text or NA)*

In your experience, what have been the main **benefits** of **in-person global health electives/experiences?** *Please describe the benefits and explain why you think these are the main benefits for you. (free text or NA)*

22. In your experience, what have been the main **concerns and challenges** of **in-person global health electives/experiences?** *Please describe the concerns and challenges and explain why you think these are the main concerns and challenges for you. (free text or NA)*

23. Complete Part C-3

Part C-3: Collaborative Global Health Activities

Collaborative global health activities are defined as activities in which members of both/several sides of a global health partnership(s) are involved in the preparation or presentation of the activity, and ideally members from both/several sides of a partnership attend and actively participate in the activity in real time.

I have/had access to (or participants at my organization have/had access to)… (check all that apply)

Activity	**Led by my organization**				**Led by another organization**				**N/A** (Check this box if you did not have access to the activity)	**Don't know**
	**Pre-pandemic *(before March 2020)***		**During pandemic *(after March 2020)***		**Pre-pandemic *(before March 2020)***		**During pandemic *(after March 2020)***			
	**In person**	**virtual**	**In person**	**virtual**	**In person**	**virtual**	**In person**	**virtual**		
collaborative global health education sessions between my organization and a global health partner(s)										
collaborative ward, clinical or laboratory rounds										
collaborative clinical case support activities (i.e., case discussions, discussions with specialists, etc.)										
collaborative research activities (i.e., literature reviews, project proposals, review board submissions,										

protocol writing, publications etc.)										

Note: The Following questions will be asked once per Part C subsection

We now will ask you about your general experience with **collaborative global health activities.**

24. In your experience, what have been the main **benefits** of **virtual collaborative global health activities?** *Please describe the benefits and explain why you think these are the main benefits for you. (free text or NA)*
25. In your experience, what have been the main **concerns and challenges** of **virtual collaborative global health activities?** *Please describe the concerns and challenges and explain why you think these are the main concerns and challenges for you. (free text or NA)*
26. In your experience, what have been the main **benefits** of **in person collaborative global health activities?** *Please describe the benefits and explain why you think these are the main benefits for you. (free text or NA)*
27. In your experience, what have been the main **concerns and challenges** of **in person collaborative global health activities?** *Please describe the concerns and challenges and explain why you think these are the main concerns and challenges for you. (free text or NA)*

28. Complete Part C-4

Part C-4: Access to global health professional development

I have/had access to (or participants at my organization have/had access to)… (check all that apply)

<table>
<tr><th rowspan="3">Activity</th><th colspan="4">At my organization</th><th colspan="4">At another organization</th><th rowspan="3">N/A
(Check this box if you did not have access to the activity)</th><th rowspan="3">Don’t know</th></tr>
<tr><th colspan="2">Pre-pandemic (before March 2020)</th><th colspan="2">During pandemic (after March 2020)</th><th colspan="2">Pre-pandemic (before March 2020)</th><th colspan="2">During pandemic (after March 2020)</th></tr>
<tr><th>In person</th><th>virtual</th><th>In person</th><th>virtual</th><th>In person</th><th>virtual</th><th>In person</th><th>virtual</th></tr>
<tr><td>global health mentorship (i.e., for career development, scholarly activity guidance, etc.)</td><td></td><td></td><td></td><td></td><td></td><td></td><td></td><td></td><td></td><td></td></tr>
<tr><td>academic recognition for global health activities (i.e., activities are eligible for academic credit, count towards a global health pathway/track/certificate</td><td></td><td></td><td></td><td></td><td></td><td></td><td></td><td></td><td></td><td></td></tr>
</table>

program, or are considered for promotion)										
global health networking activities (i.e., conferences, meetings, etc.) to connect with other professionals										

Note: The Following questions will be asked once per Part C subsection

We now will ask you about your general experience with **global health professional development.**

29. In your experience, what have been the main **benefits** of **virtual global health professional development?** *Please describe the benefits and explain why you think these are the main benefits for you. (free text or N/A)*
30. In your experience, what have been the main **concerns and challenges** of **virtual global health professional development?** *Please describe the concerns and challenges and explain why you think these are the main concerns and challenges for you.(free text or N/A)*
31. In your experience, what have been the main **benefits** of **in person global health professional development?** *Please describe the benefits and explain why you think these are the main benefits for you.(free text or N/A)*
32. In your experience, what have been the main **concerns and challenges** of **in person global health professional development?** *Please describe the concerns and challenges and explain why you think these are the main concerns and challenges for you.(free text or N/A)*

Part D. Benefits and Challenges to virtual global health activities (VGHAs)
(Addresses OBJECTIVE 3 & 5)

33. In your opinion what is the **greatest potential benefit** of virtual global health activities? *(choose one)*

a. Complement existing global health activities at my organization
b. Be useful even after in-person activities resume
c. Improve access to resources (i.e., journals, subscriptions, curricula, etc.)
d. Improve access to clinical opportunities
e. Improve access to professional support/mentorship
f. Improve ability to network with professional colleagues
g. Maintain global health partnerships over time
h. Improve bidirectionality within partnerships
i. Improve equity within global health partnerships
j. Improve IT/tech capacity within my organization
k. Reach more learners than otherwise possible
l. Allow safer educational endeavors
m. Allow for career advancement
n. Allow for less-costly global health education activities
o. Avoid burnout
p. Other, please specify *(free text)*

34. In your opinion what is the **greatest potential challenge** of virtual global health activities? *(choose one)*

a. Cost of training faculty/staff/learners
b. Time needed to train faculty/staff/learners
c. Lack of space/facilities that could be dedicated to virtual activities
d. Lack of institutional support (financial, policy, etc.)
e. Lack of equipment to be used in virtual activities
f. Lack of formal training curriculum
g. Lack of necessary wifi bandwidth or resources to cover wifi cost
h. Lack of mentors/local champions at my site
i. Lack of consistent or reliable internet access
j. Lack of knowledge or skills in developing and/or implementing virtual activities
k. Difficulty retaining and working with interested learners
l. Lack of equal experiences between partners
m. Other, please specify *(free text)*

Part E. Interest in Virtual Global Health Activities
(Addresses OBJECTIVE 4 & 5)

35. Complete the following table

Interest in Virtual Activities
(Please select one answer per row)

Are you interested in the following activities virtually?

Virtual Global Health Activity	Yes, during pandemic but not after	Yes, after pandemic but not during	Yes, during AND after pandemic	Not interested
professional resources (i.e., library services, access to journals/publications, online subscription services, etc.) that support global health activities				
global health educational materials (i.e., curricula, textbooks, modules, etc.)				
global health didactic sessions (i.e., grand rounds, case discussions or journal clubs, etc.)				
global health simulation sessions (i.e., clinical, procedural, or cultural simulations)				
global health experience preparation sessions (i.e., pre-departure trainings, global health boot camps, cultural trainings, etc.)				
**local** global health experiences				
**Domestic/national** global health experiences				
**international** global health experiences				
Hosting participants from outside my organization				
collaborative global health education sessions between my organization and a global health partner(s)				
collaborative ward, clinical or laboratory rounds				
collaborative clinical case support activities (i.e., case discussions, discussions with specialists, etc.)				

collaborative research activities (i.e., literature reviews, project proposals, review board submissions, protocol writing, publications etc.)				
global health mentorship (i.e., for career development, scholarly activity guidance, etc.)				
academic recognition for global health activities (i.e., activities are eligible for academic credit, count towards a global health pathway/track/certificate program, or are considered for promotion)				
global health networking activities (i.e., conferences, meetings, etc.) to connect with other professionals				

36. We'd like to hear your opinions about how to improve the quality of ***virtual*** global health activity(ies). Please provide your suggestions in the box below: *(free text)*

END SURVEY

Thank you for completing this survey.

If you are interested in participating in a voluntary raffle for one of five Amazon gift cards, please click 'submit' below. You will be taken to a new page to enter your email address.

The raffle is optional. We will not store or later access any personal information you provide to participate in the raffle, and your email address will not be linked to your survey responses.

Please contact the study Principal Investigator with any questions.
