# Supplementary figures and images for: Income-Based Disparities in Perceived Benefits and Challenges of Virtual Global Health Activities During the COVID-19 Pandemic: Mixed Methods Analysis
Source: J Med Internet Res. 2025 May 7;27:e63066. doi: 10.2196/63066 (PMC12096022; doi:10.2196/63066)

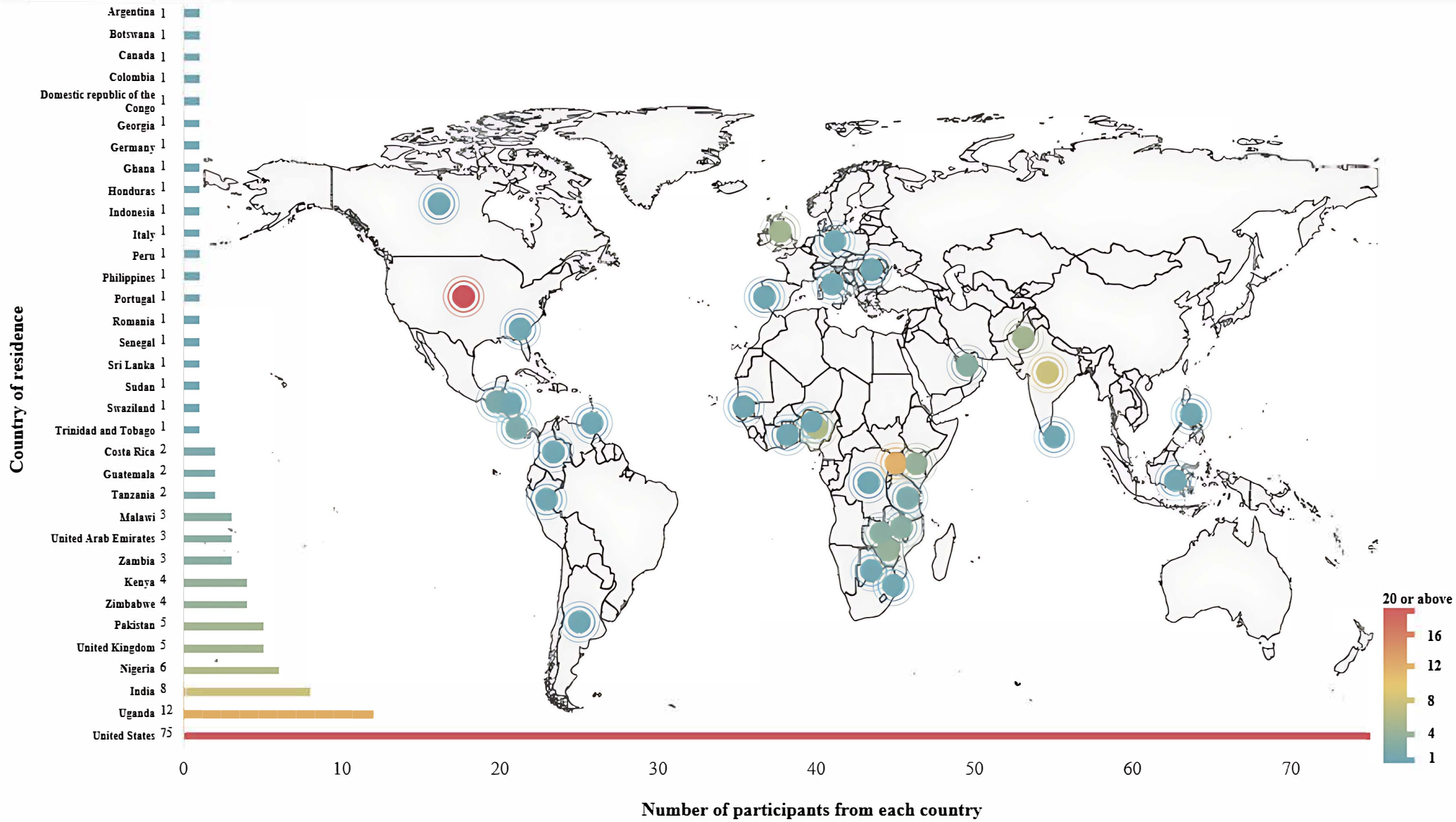

Supplement: Multimedia Appendix 3 [file jmir_v27i1e63066_app3.pdf]
