# Supplementary material for: Income-Based Disparities in Perceived Benefits and Challenges of Virtual Global Health Activities During the COVID-19 Pandemic: Mixed Methods Analysis
Source: J Med Internet Res. 2025 May 7;27:e63066. doi: 10.2196/63066 (PMC12096022; doi:10.2196/63066)
Supplement: Multimedia Appendix 4 [file jmir_v27i1e63066_app4.docx]

**Multimedia Appendix 4**

A summary of themes on perceived benefits and challenges of in-person global health activities stratified by low- and middle-income country and high-income country respondents.^a^

| Themes | | Quotes | LMIC^b^ respondents who mentioned the corresponding theme (n=67), n (%) | HIC^c^ respondents who mentioned the corresponding theme (n=87), n (%) | *P* value^d^ |
| --- | --- | --- | --- | --- | --- |
| **Perceived benefits of in-person GHAs^e^** | | | | | |
|  | Positive in-person experiences | - “Developing personal connection and being able to share more of the cultural experience and appreciation than virtual seminars and learning sessions.” [Participant 233, LMIC] - “In-person also allows more intensive application of new resources to your own work and settings. Without the in-person application, the virtual models are limited in value.” [Participant 230, HIC] | *30 (45)* | *60 (69)* | *.003* |
|  | Easier networking, new relationships, or activities | - “The interpersonal connections are much stronger (especially when building new relationships, which can be hard to do over Zoom).” [Participant 31, HIC] - “Meeting people in person helps to cement relationships and with the extra time additional opportunities for collaboration and networking usually present themselves.” [Participant 43, LMIC] | *20 (30)* | *45 (52)* | *.007* |
|  | Improved communication and collaboration | - “They are so much richer. Virtual meetings are usually time limited for a variety of reasons. In person meetings can always be lengthened or social activities arrange to extend discussions.” [Participant 93, HIC] | 15 (22) | 21 (24) | .80 |
|  | Improved knowledge and skills | - “Being able to travel and experience global health in person has continued to inspire me to pursue a career in global health. Attending to medical issues not often seen in one’s home country and learning about different health care systems is a valuable experience to contribute to one’s knowledge.” [Participant 142, HIC] - “It provides a more memorable and practical experience compared to the theoretical approach of virtual.” [Participant 43, LMIC] | 17 (25) | 14 (16) | .15 |
|  | Improved activity efficiency | - “Same time zone, able to interact using body language, socialize, build trust, establish relationships, more productive and more efficient use of time.” [Participant 61, HIC] - “Lesser distractions when I access a resource in person compared to accessing a resource online.” [Participant 209, LMIC] | 12 (18) | 17 (20) | .80 |
|  | Improved interest or engagement in GH^f^ | - “Greater interest in global problems and solutions.” [Participant 21, LMIC] - “Being able to gain a really good grasp of what is going on in-country. To also work with partners to share you own understanding of a situation which can be enriched by working with an overseas’ colleague by actually being there.” [Participant 93, HIC] | *3 (4)* | *14 (16)* | *.02* |
|  | Easier continuity of relationships and activities | - “For the hosting institution, establishing long term partnerships with institutions in high resource settings is certainly valuable, and [it] improves the health services available at the hosting institutions.” [Participant 91, LMIC] - “A ‘reward’ for a longer-term connection/collaboration, networking, the intangibles of learning together, camaraderie, used as a platform for longer-term connections, gain a deeper understanding of resources/needs/culture.” [Participant 95, HIC] | 4 (6) | 8 (9) | .46 |
|  | More real-world impacts | - “The main benefits are help the disadvantage child, girls and women on community.” [Participant 159, LMIC] | 6 (9) | 6 (7) | .64 |
|  | Easy scheduling or planning | - “Ability to have more frequent sessions between our trainees and international trainees.” [Participant 124, LMIC] - “Chances of leaving a meeting abruptly to attend other business are low as compared to what can be done online.” [Participant 179, LMIC] | 4 (6) | 2 (2) | .24 |
|  | Improved support for learners or faculty | - “We have the opportunity to spend more time with our colleagues outside our organization and also access more support materials for research.” [Participant 216, LMIC] | 1 (1) | 5 (6) | .18 |
| ***Perceived challenges of in-person GHAs*** | | | | | |
|  | High cost | - “High costs of travel, accommodation and registration fees: this is an important issue especially for those mainly from [LMICs] who can’t afford those costs and may have no voice in the global arena.” [Participant 10, HIC] - “Per diems are a large challenge to in person collaborative global health activities in this setting—it is often impossible to have a collaborative activity without a large budget for per diems for participants.” [Participant 91, LMIC] | 32 (48) | 33 (38) | .22 |
|  | COVID-19 pandemic-related concerns for engagement | - “Fear of becoming sick in a developing country with little to no resources. Fear and uncertainty about COVID and lack of infrastructure and vaccination in countries.” [Participant 104, LMIC] | 19 (28) | 35 (40) | .13 |
|  | Requires travel to sites | - “The time it takes to actually go to in-person events. From driving to parking to finding the room to getting back home is probably in the neighborhood of an hour and a half per session, which is a lot of time that’s effectively wasted.” [Participant 180, HIC] - “Travel has obviously become more difficult during the pandemic.” [Participant 91, LMIC] | 17 (25) | 24 (28) | .76 |
|  | Difficulty in scheduling or planning | - “Schedule constraints [the ability to] travel for in-person experiences.” [Participant 31, HIC] - “Time is very limited sometimes I miss this section because of shortage of time to attend.” [Participant 107, LMIC] | *11 (16)* | *26 (30)* | *.05* |
|  | Inequitable GH engagement | - “(Mainly from emerging countries) who can't afford those costs and may have no voice in the global arena.” (Participant 10, HIC) - “Inequitable distribution of some of these resources have been a major challenge.” [Participant 23, LMIC] | 10 (15) | 10 (11) | .53 |
|  | Challenging to engage on-site | - “Only specific types of international work allowed.” [Participant 49, HIC] - “Fragmented health infrastructure.” [Participant 130, HIC] - “Some conferences made more provision for virtual attendance than for in-person attendance which may make one attend a conference virtually.” [Participant 216, LMIC] | 10 (15) | 17 (20) | .46 |
|  | Communication challenges | - “Language—not everything is translated into English from Kinyarwandan.” [Participant 255, HIC] - “Many resources are only in English.” [Participant 241, LMIC] | 4 (6) | 5 (6) | .95 |
|  | Lack institutional support or awareness | - “Lack of institutional support.” [Participant 4, HIC] | 0 (0) | 2 (2) | .21 |

^a^Pearson *χ*^2^ tests were conducted to examine whether the frequency of mentioning identified themes varies between respondents living in HICs and LMICs.

^b^LMIC: low- and middle-income country.

^c^HIC: high-income country.

^d^Themes with significant differences (*P* ≤.05) in the frequency of mentioning between LMIC and HIC respondents are italicized.

^e^GHA: global health activity.

^f^GH: global health.
