# Supplementary material for: Income-Based Disparities in Perceived Benefits and Challenges of Virtual Global Health Activities During the COVID-19 Pandemic: Mixed Methods Analysis
Source: J Med Internet Res. 2025 May 7;27:e63066. doi: 10.2196/63066 (PMC12096022; doi:10.2196/63066)
Supplement: Multimedia Appendix 5 [file jmir_v27i1e63066_app5.docx]

**Multimedia Appendix 5**

A summary of themes on perceived benefits and challenges of virtual global health activities stratified by low- and middle-income country and high-income country respondents.^a^

| Themes | | Quotes | LMIC^b^ respondents who mentioned the corresponding theme (n=67), n (%) | HIC^c^ respondents who mentioned the corresponding theme (n=87), n (%) | *P* value^d^ |
| --- | --- | --- | --- | --- | --- |
| **Perceived benefits of VGHAs^e^** | | | | | |
|  | Improves access to GH^f^ resources or content | - “Shared access to materials and experts that are not available in my organization.” [Participant 126, HIC] - “Access to coursework from anywhere at any time.” [Participant 97, LMIC] | 49 (73) | 52 (60) | .08 |
|  | Reduces cost | - “Many conference organizers have made their virtual conferences either freely available or at reduced costs to participants from low-resource settings.” [Participant 93, HIC] - “Reduce travelling costs and other related expenses. Making them more accessible to participant with limited incomes.” [Participant 241, LMIC] | *26 (39)* | *20 (23)* | *.03* |
|  | Enables remote participation | - “Easier than in-person activities because I don’t have to consider transportation or extra time to go to and from the activity. Able to do the activities from home, which can be better for my mental health when I don’t get to spend much time at home.” [Participant 85, HIC] | 15 (22) | 28 (32) | .18 |
|  | Fosters easy scheduling and planning | - “Most virtual activities can be done during your free time so it does not take time away from work.” [Participant 43, LMIC] | 14 (21) | 25 (29) | .27 |
|  | Wider participation and reach | - “The main benefits are that the virtual platform can connect facilities that are geographically distant from one another. For instance, in TZ, the national pediatric oncology program has a weekly teleconference with referring hospitals to discuss management of all pediatric patients with cancer. This collaboration has greatly expanded the availability of pediatric cancer services across the nation. And virtual collaborative global health activities are especially helpful for locations that are geographically remote so may not be able to meet easily with other partners in person.” [Participant 91, LMIC] | 11 (16) | 25 (29) | .08 |
|  | Fosters networking and new relationships or activities | - “Virtual meetings have allowed the team to gather more frequently. Also, new local and international members have been able to join easier to the team.” [Participant 241, LMIC] - “We were able to begin Telehealth with our partners in Guatemala. In addition, we will be able to continue telehealth expanding how we can meet outpatient needs for our partners even after we go back to in person experiences.” [Participant 96, HIC] | 11 (16) | 23 (26) | .14 |
|  | Fosters continuity of relationships or activities despite the pandemic | - “The ability to continue GH activities during the pandemic was only possible via virtual media, and the opportunity to continue those activities was invaluable.” [Participant 31, HIC] | *6 (9)* | *28 (32)* | *<.001* |
|  | Fosters easy participation and engagement | - “Combination of structure and freedom, effective time management, expanded world view, asynchronous discussions with participants, immediate feedback on questions.” [Participant 97, LMIC] - “Within an established research or collaborative partnership, virtual is faster, more continuous and feels more organic than waiting for in-person engagements. Especially since it is now normalized—in many instances virtual engagements used to feel more like a replacement or a place holder.” [Participant 230, HIC] | 14 (21) | 12 (14) | .24 |
|  | Expands knowledge, experience, or skills | - “Practitioner have shared various experiences and thus benefiting people around the world.” [Participant 76, LMIC] - “Being able to teach virtually to audiences around the world.” [Participant 239, LMIC] | *15 (22)* | *8 (9)* | *.02* |
|  | Improves collaboration, communication, and relationships | - “Increased access and frequency of communication with global partners. Improved telecommunication platforms (zoom, teams) have decreased barriers.” [Participant 52, HIC] | 6 (9) | 12 (14) | .35 |
|  | More equity | - “More equal voices from low resource partners.” [Participant 4, HIC] - “Collaboration with people from all over the world and better opportunities to share resources and encourage voicing differing opinions.” [Participant 233, LMIC] | 3 (4) | 8 (9) | .26 |
|  | Improved clinical and patient outcomes | - “Improve therapy related outcome, this is because latest issues on drug use and pharmaceutical technology.” [Participant 158, LMIC] | 2 (3) | 0 (0) | .11 |

| **Perceived Challenges of VGHAs** |
| --- |

|  | Lack of infrastructure to engage virtually | - “We have had a mix of meetings where some partners have been able to remain online throughout the activity and enjoyed the whole experience and others (mainly those from low-resource settings) who do not have the benefit of strong bandwidth who have either not been able to join in activities or had partial access to an activity and therefore not been able to access the whole picture.” [Participant 93, HIC] - “Data challenges ad poor power supply.” [Participant 240, LMIC] - “Limited access to the reliable internet connections especially in the remote areas that we operate in.” [Participant 76, LMIC] | *38 (57)* | *31 (36)* | *.009* |
| --- | --- | --- | --- | --- | --- |
|  | Less engaged and motivated to participate virtually | - “I think people are somewhat less engaged and excited about preparing a presentation for a virtual event. This is true for me, so I haven’t proposed anything since the pandemic. I am not as interested in sitting in my office listening to a talk on a computer as I would be a live event.” [Participant 6, HIC] - “Screen time is more health issues from sitting in the same spot for too long.” [Participant 240, LMIC] | *19 (28)* | *43 (49)* | *.008* |
|  | Lack of in-person and hands-on experience | - “It is just not the same as meeting/talking/learning in person...not as enriching. But better than nothing. I have ‘met’ and created relationships with people virtually during the pandemic, which was great, but it was so much better finally to meet those people in person when I traveled to Liberia at the end of 2021.” [Participant 134, HIC] - “It is harder to fully understand the landscape in another country without actually being there. It’s still great to discuss issues that happen globally, but there is a missing element when you are not on the ground there.” [Participant 21, HIC] - “The main challenge with virtual access is not being able to engage directly with colleagues. Virtual meetings are helpful, but cannot replicate the connections made with in person meetings.” [Participant 58, LMIC] | 23 (34) | 42 (48) | .08 |
|  | Difficult scheduling or planning | - “The inability of meeting at the same time during 2020 had 2 studies stopped because the radiologists involved at Children’s and the International partner had difficult schedules.” [Participant 171, HIC] - “Countries have different time zones makes it difficult.” [Participant 34, LMIC] | 17 (25) | 28 (32) | .36 |
|  | Difficult to network or build new relationships virtually | - “The inability to travel to a research area and moreover to lose a lot of that experience by working with colleagues with whom working relationships had already been formed and the inability to work with new colleagues.” [Participant 93, HIC] | 10 (15) | 23 (26) | .08 |
|  | Challenges with virtual communication and collaboration | - “Students are often hung up on logistics and minor issues (difficult to hear/interpret accents and dialects when not in person, harder to read body language especially in new cultural setting).” [Participant 230, HIC] - “Conversation can easily become one-sided and sometimes the fake news promoters (especially in the case of COVID vaccination campaign) can overwhelm the social media and shun those who disagree with the arguments.” [Participant 233, LMIC] | 11 (16) | 15 (17) | .89 |
|  | High cost to participate and lack of funding | - “Data is expensive in our country and after working hours one can miss a session they may want to attend.” [Participant 179, LMIC] - “Our budget was also cut tremendously due to COVID.” [Participant 128, HIC] | *13 (19)* | *6 (7)* | *.02* |
|  | GH resources or content hard to access virtually | - “May not be able to access all learning materials from our counterparts abroad.” [Participant 216, LMIC] - “Publication access in LMIC remains an issue.” [Participant 45, HIC] | 3 (4) | 8 (9) | .26 |
|  | Lack of support to learners or faculty | - “Not able to have informal meetings and in person coaching.” [Participant 78, HIC] - “Lack of collaboration with the course holders.” [Participant 26, LMIC] | 3 (4) | 4 (5) | .97 |
|  | COVID-19 pandemic slowed virtual programs | - “Almost all global health opportunities shut down following the onset of the pandemic. It’s not so much that these resources aren’t available, but rather that there are minimal current opportunities to participate.” [Participant 180, HIC] | 2 (3) | 5 (6) | .42 |
|  | Knowledge gaps making virtual participation difficult | - “Not many people are literate enough to have access to evidence-based health resources (especially for those who live in the rural areas and the elderly).” [Participant 233, LMIC] | 3 (4) | 2 (2) | .45 |
|  | Difficult to maintain relationships or activities virtually | - “The inability to travel to a research area and moreover to lose a lot of that experience by working with colleagues with whom working relationships had already been formed and the inability to work with new colleagues.” [Participant 93, HIC] | 2 (3) | 3 (3) | .87 |
|  | Less real-world impact | - “Less impactful than in-person.” [Participant 45, HIC] | 0 (0) | 4 (5) | .08 |
|  | Reduced knowledge or skills learned | - “Hard to explain procedures that need in-person explanations and simulations which limits the topics that can be discussed.” [Participant 241, LMIC] | 1 (1) | 3 (3) | .45 |

^a^Pearson *χ*^2^ tests were conducted to examine whether the frequency of mentioning identified themes varies between respondents living in HICs and LMICs.

^b^LMIC: low- and middle-income country.

^c^HIC: high-income country.

^d^Themes with significant differences (*P* ≤.05) in the frequency of mentioning between LMIC and HIC respondents are italicized.

^e^VGHA: virtual global health activity.

^f^GH: global health.
