# Supplementary material for: Income-Based Disparities in Perceived Benefits and Challenges of Virtual Global Health Activities During the COVID-19 Pandemic: Mixed Methods Analysis
Source: J Med Internet Res. 2025 May 7;27:e63066. doi: 10.2196/63066 (PMC12096022; doi:10.2196/63066)
Supplement: Multimedia Appendix 6 [file jmir_v27i1e63066_app6.docx]

**Multimedia Appendix 6**

A summary of themes on suggestions for improving the quality of VGHAs stratified by low- and middle-income country and high-income country respondents.^a^

| Themes | Quotes | LMIC^b^ respondents who mentioned the corresponding theme (n=67), n (%) | HIC^c^ respondents who mentioned the corresponding theme (n=87), n (%) | *P* value^d^ |
| --- | --- | --- | --- | --- |
| Making participation as easy and equitable as possible | “To diversify on the type of languages used because different people know different languages according to their tribes especially in the low and middle-income countries.” [Participant 121, LMIC] | 9 (13) | 5 (6) | .10 |
| Consider resource availability | “The first thing is to consider the issue of bandwidth. At times, participants have struggled to participate in virtual sessions because of connection challenges. Providing resources for this challenge could prove to be very vital.” [Participant 76, LMIC] | 5 (7) | 6 (7) | .89 |
| Invest in training for VGHAs^e^ | “We should have skills in using video conference tools effectively.” [Participant 145, LMIC] | 6 (9) | 3 (3) | .15 |
| Providing guidelines for VGHAs and better engagement | “Having some suggested approaches in terms of frequency of meetings and approach during early partnerships—templates—might be useful for all parties to clearly understand.” [Participant 151, HIC] | 6 (9) | 2 (2) | .07 |
| Making content as interesting and relevant as possible | “Ask for subject submissions. Many of the perceived topics of interest aren’t really the ones most needing at international sites. Make them available for medical students that have global health interests in developing countries—for them it’s everyday medicine.” [Participant 104, LMIC] | *6 (9)* | *1 (1)* | *.02* |
| Combining virtual with in-person activities | “When able, offer hybrid opportunities and opportunities for casual connections as well. Each time I come lecture for your GH symposia the most meaningful moments were with participants who come to talk with me about global mental health after my talks. This gets lost in virtual only.” [Participant 52, HIC] | 2 (3) | 2 (2) | .79 |
| Improving institutional support and recognition of VGHAs | “I’m not sure that academic institutions understand the value of VGHAs. That means they are not recognized for faculty promotion, no time/resources are devoted to them, and I think it’s up to individuals or smaller programs to ‘do the right thing.’ It means that collaborations are vulnerable to a handful of individuals losing time/interest, or moving on to a new position. If the quality is to improve, there needs to be institutional buy-in.” [Participant 95, HIC] | 1 (1) | 3 (3) | .45 |
| Pursuing collaborations for VGHAs | “Using the opportunity to incorporate people from across organizations and other countries.” [Participant 141, HIC] | 2 (3) | 1 (1) | .41 |

^a^Pearson *χ*^2^ tests were conducted to examine whether the frequency of mentioning identified themes varies between respondents living in HICs and LMICs.

^b^LMIC: low- and middle-income country.

^c^HIC: high-income country.

^d^Themes with significant differences (*P* ≤.05) in the frequency of mentioning between LMIC and HIC respondents are italicized.

^e^VGHA: virtual global health activity.
